# *Infectious Agents and Cancer* reviewer acknowledgement 2013

**DOI:** 10.1186/1750-9378-9-5

**Published:** 2014-01-31

**Authors:** Franco M Buonaguro, Sam M Mbulaiteye

**Affiliations:** 1 Istituto Nazionale Tumori, Naples, Italy; 2 National Cancer Institute, Bethesda, USA

## Abstract

**Contributing reviewers:**

The editor of *Infectious Agents and Cancer* would like to thank all our reviewers who have contributed to the journal in Volume 32 (2013).

## 

Clement Adebamowo

United States of America

Marla Amarante

Brazil

Koofe Anaa

Nigeria

Cote Velez Antonieta

Mexico

Ramandeep Arora

India

Birgitta Asjo

Norway

Kari Auranen

Finland

Amaya Ayaka

Japan

Leona W Ayers

United States of America

Ramy Karam Aziz

Egypt

Cecily Banura

Uganda

Ruanne Barnabas

United States of America

Jaime Berumen

Mexico

Kishor Bhatia

United States of America

Jorge Blanco

Spain

Florentina Bocaneti

Romania

Kocjan Bostjan

Slovenia

Sabine Brandt

Austria

Andréia Buffon

Brazil

Christine Bundell

Australia

Pierre Busson

France

Raleigh Butler

Bahamas

Yu-Sun Chang

Taiwan

Jen-Yang Chen

Taiwan

Wuguo Chen

United States of America

Brian Cox

New Zealand

Bingbing Dai

United States of America

Chao Dai

United States of America

Luigino Dal Maso

Italy

Antonella De Luca

Italy

Valli De Re

Italy

Silvia De Sanjose

Spain

Paola Di Bonito

Italy

Vito Di Lernia

Italy

Elizabeth Didier

United States of America

Cyril Dim

Norway

Cyril Dim

Nigeria

Silvya Engler

Brazil

Nicholas Eyre

Australia

María Dolores Fellner

Argentina

Catterina Ferreccio

Chile

Renato Franco

Italy

Bin Gao

China

Magda Garzón

Spain

Yong Song Gho

Korea, South

Paolo Giorgi Rossi

Italy

Patricia Goggin

Canada

Satish Gopal

Malawi

Martin Hale

South Africa

Russell Hales

United States of America

Diane Harper

United States of America

Charlotte Haug

Norway

Fum Haya

Japan

Pengiran Hishamuddin

Singapore

Yeung Ho

United States of America

Markus Hoffmann

Germany

Myfanwy Hopkins

United States of America

Syed Hussain

Pakistan

Rs Jayshree

India

Rajiv Khanna

Australia

Bodin Khwannimit

Thailand

James Kitinya

Tanzania

Indu Kohaar

United States of America

Andrei Kozlov

Russian Federation

Chengyu Liang

United States of America

Flavia Lillo

Italy

David Lindquist

Sweden

Kwok Wai Lo

Hong Kong

Rubén López-Revilla

Mexico

Koanga Mogtomo Martin Luther

Cameroon

Koji Matsumoto

Japan

Jaap M. Middeldorp

Netherlands

Mona Mohamed

Egypt

Denis Mottet

Belgium

Robert Newton

United Kingdom

Mohammad Nickdel

United Kingdom

Shuji Ogino

United States of America

Luigi Panico

Italy

Petros Papagerakis

United States of America

Wilson Parawira

Zimbabwe

Pragna Patel

United States of America

Eduardo Perez Salazar

Mexico

Annacarmen Petrizzo

Italy

Beatriz Pogo

United States of America

Camillo Porta

Italy

Arnold Rabson

United States of America

Ribas-Aparicio Rosa Maria

Mexico

Martin Rowe

United Kingdom

Marco Ruiz

United States of America

Mauricio Salcedo

Mexico

Lars Sand

Sweden

Shahin Sayed

Kenya

Abdul Moid Shehzad

Pakistan

Maria Angélica Silva

Brazil

Elaine Smith

United States of America

Jean Pierre Spinosa

Switzerland

Marc Steben

Canada

Charles Stiller

United Kingdom

Rosamaria Tedeschi

Italy

Lele Terada

United Kingdom

Andrea Tinelli

Italy

Maria Lina Tornesello

Italy

Joseph Tota

Canada

Mounir Trimeche

Tunisia

Anastasios Tsaousis

United Kingdom

Edda Vuhahula

Tanzania

Xingmin Wang

United States of America

Maria Angelica Ehara Watanabe

Brazil

Justin Weir

United Kingdom

Shih-Hsiung Wu

Taiwan

Chezion Xen

China

Jianfei Xue

United States of America

Badrul Yahaya

Malaysia

Yibin Yang

United States of America

Lee Fah Yap

Malaysia

Chen Zhao

United States of America

